# RAF1 promotes lymphatic metastasis of hypopharyngeal carcinoma via regulating LAGE1: an experimental research

**DOI:** 10.1186/s12967-022-03468-7

**Published:** 2022-06-06

**Authors:** Yanshi Li, Min Pan, Tao Lu, Dan Yu, Chuan Liu, Zhihai Wang, Guohua Hu

**Affiliations:** Department of Otorhinolaryngology, The First Affiliated Hospital of Chongqing Medical University, Chongqing, 400016 China

**Keywords:** Hypopharyngeal carcinoma, Lymphatic metastasis, Transcriptome sequencing, Proteomic sequencing, RAF1, LAGE1

## Abstract

**Background:**

Lymphatic metastasis was an independent prognostic risk factor for hypopharyngeal carcinoma and was the main cause of treatment failure. The purpose of this study was to screen the differential genes and investigate the mechanism of lymphatic metastasis in hypopharyngeal carcinoma.

**Methods:**

Transcriptome sequencing was performed on primary tumors of patients, and differential genes were screened by bioinformatics analysis. The expression of differential genes was verified by qRT-PCR, western-blotting and immunohistochemical, and prognostic value was analyzed by Kaplan–Meier and log-rank test and Cox’s test. Next, FADU and SCC15 cell lines were used to demonstrate the function of differential genes both in vitro by EdU, Flow cytometry, Wound Healing and Transwell assays and in vivo by a foot-pad xenograft mice model. Proteomic sequencing was performed to screen relevant targets. In addition, in vitro and in vivo experiments were conducted to verify the mechanism of lymphatic metastasis.

**Results:**

Results of transcriptome sequencing showed that RAF1 was a significantly differential gene in lymphatic metastasis and was an independent prognostic risk factor. In vitro experiments suggested that decreased expression of RAF1 could inhibit proliferation, migration and invasion of tumor cells and promote apoptosis. In vivo experiments indicated that RAF1 could promote tumor growth and lymphatic metastasis. Proteomic sequencing and subsequent experiments suggested that LAGE1 could promote development of tumor and lymphatic metastasis, and was regulated by RAF1.

**Conclusions:**

It suggests that RAF1 can promote lymphatic metastasis of hypopharyngeal carcinoma by regulating LAGE1, and provide a basis for the exploring of novel therapeutic target and ultimately provide new guidance for the establishment of intelligent diagnosis and precise treatment of hypopharyngeal carcinoma.

## Background

Head and neck tumors are the sixth most common malignant tumors worldwide, of which more than 90% are squamous cell carcinoma, collectively known as head and neck squamous cell carcinoma (HNSCC). In recent years, although the study of this disease and the comprehensive treatment such as surgery, radiotherapy and chemotherapy, targeted therapy and immunotherapy have made some progress, the prognosis of patients is still poor, and the 5-year survival rate is only about 50% [1–6]. Hypopharyngeal carcinoma is one of the most malignant tumors of HNSCC, accounting for 0.8–1.5% of malignant tumors of HNSCC [7]. In recent years, the incidence of hypopharyngeal carcinoma is on the rise, and there is a younger trend [8]. Due to the concealed anatomical structure of hypopharynx, the early clinical symptoms are not obvious, and it is easy to misdiagnosis. When significant clinical symptoms occur and the patients are admitted to the hospital, they are already in the advanced stage. Meanwhile, hypopharyngeal carcinoma is highly heterogeneous and prone to cervical lymph node metastasis or invasion of adjacent sites [9–11]. At present, the main clinical treatment for hypopharyngeal carcinoma is conservative or radical surgery supplemented with preoperative concurrent chemoradiotherapy and postoperative complementary chemoradiotherapy. However, the five-year postoperative survival rate of hypopharyngeal carcinoma patients is 25–40%, and the survival rate of patients with radiotherapy and chemotherapy alone is only 12–14% [12–14].

Malignant tumor metastasis is an important factor affecting the choice of treatment regimen, treatment effect and survival time of patients. Compared with hematogenous metastasis, there are few studies on the mechanism of lymphatic metastasis. Cervical lymph node metastasis is an independent risk factor for prognosis of hypopharyngeal cancer. Lymph node diameter, number of metastatic lymph nodes and extracapsular spread all affect the prognosis of hypopharyngeal carcinoma, and the existence of lymphatic metastasis reduces the overall survival rate by nearly 50% [15–17]. Lymphatic metastasis is a complex pathologic process, which mainly includes the growth and invasion of tumor cells, lymphangiogenic cytokines secretion and lymphangiogenesis [18–20]. Recent studies have shown that vascular endothelial growth factor-C (VEGF-C), lymphatic vessel endothelial hyaluronan receptor 1 (LYVE-1), and prospero homeobox protein 1 (PROX-1) are common lymphangiogenic cytokines, and their expression levels are closely related to lymphangiogenesis [21–24]. Therefore, it is of great significance to explore the molecular markers and mechanisms of lymphatic metastasis of hypopharyngeal carcinoma for clinical work and prognosis of patients. The elucidation of this mechanism will provide new ideas for early clinical diagnosis and treatment. On the one hand, it can standardize the scope and degree of lymph node dissection in surgical treatment of hypopharyngeal carcinoma, and on the other hand, it can provide new guidance for targeted therapy of tumors from the perspective of blocking signal pathways and antagonizing relevant effecting molecules.

Multi-omics sequencing mainly includes genome sequencing, transcriptome sequencing, proteomics sequencing and metabonomics sequencing. It is a mature, efficient and high-throughput detection method, which can be used to verify cancer biomarkers, explore gene functions and develop novel targeted drugs [25–27]. Genomic and transcriptomic studies have revealed that HNSCC is a highly heterogeneous malignant tumor, which is closely related to tumor metastasis and drug resistance [28, 29]. Recent advances in single-cell transcriptomics have provided avenues to explore tumor heterogeneity and tumor microenvironment at the single-cell level, revealing new insights into tumor composition, cancer stem cells, and drug resistance. In a new single-cell transcriptomic study of head and neck squamous cell carcinoma, single-cell transcriptomic maps of approximately 6000 cells from 18 primary head and neck squamous cell carcinoma and matched metastatic lymph nodes reveal the expression mechanisms that distinguish different malignant, stromal and immune cells [30]. Fortunately, in our previous studies, RAF1 has been identified as an independent prognostic risk factor for lymphatic metastasis of hypopharyngeal cancer by transcriptomic sequencing combined with clinical data [31].

Ras-associated factor -1 (RAF1) belongs to the RAF protein kinases family, also known as C-Raf. It is the cellular homolog of viral raf gene (v-raf). The encoded protein is a MAP kinase kinase kinase (MAP3K), which functions downstream of the Ras family of membrane associated GTPases to which it binds directly [32]. Once activated, the cellular RAF1 protein can phosphorylate to activate the dual specificity protein kinases MEK1 and MEK2, which in turn phosphorylate to activate the serine/threonine specific protein kinases, ERK1 and ERK2. It participates in Ras-RAF-MEK-ERk signaling pathway (MAPK signaling pathway), and transmits extracellular signals into the nucleus through cell membrane receptors, thereby mediating the expression of intracellular specific proteins and participating in the regulation of cell proliferation, differentiation, apoptosis, autophagy and other functions [33]. According to relevant reports, the high expression of RAF1 is positively correlated with the occurrence, development and clinical prognosis of a variety of cancers [34–37]. Unfortunately, the function and mechanism of RAF1 in tumor progression and lymphatic metastasis of head and neck squamous cell carcinoma has not been explored.

L Antigen Family Member 1 (LAGE1) belongs to the cancer–testis antigens family. The cancer–testis antigens family, including MAGE-A, NY-ESO-1, LAGE-1, and TTK, are potential targets for immunotherapy due to their strong immunogenicity and unique expression patterns in vivo. Cancer–testis antigens have been reported for use in cancer immunization, adoptive T cell transfer of chimeric T cell receptors, and immunity inhibitors [38–40]. In addition, up-regulation of LAGE1 expression has also been verified in various tumors [41–43]. Other studies have shown that patterns of antibody responses to nonviral cancer antigens (such as LAGE-1, MAGE-A1 etc.) are distinct based upon HPV status which can be utilized for the development of immunotherapy for HNSCC [44]. However, the main mechanism of LAGE1 and its relationship with lymphatic metastasis remain unknown.

In conclusion, as a prognostic risk factor of hypopharyngeal carcinoma, lymphatic metastasis has a great impact on the survival rate and quality of life. The purpose of this study was to screen the differential genes by multi-omics sequencing and investigate the mechanism of lymphatic metastasis in hypopharyngeal carcinoma. This study is aimed to reveal the regulatory mechanism of RAF1-LAGE1 signaling axis in modulating the evolution of lymphatic metastasis of hypopharyngeal carcinoma, and provide a basis for the exploring of novel therapeutic target, and ultimately provide new ideas for the establishment of intelligent diagnosis and precise treatment of hypopharyngeal carcinoma.

## Methods

### Patients and samples

Hypopharyngeal carcinoma tumor tissue, normal adjacent tissue and lymph node tissue were acquired from patients undergoing hypopharyngectomy in the First Affiliated Hospital of Chongqing Medical University from 2012 to 2021. The inclusion criteria were as follows: pathological diagnosis was hypopharyngeal squamous cell carcinoma; preoperative ultrasound, CT or MRI were performed to determine the extent of the lesion; hemorrhagic metastasis and distant metastasis could not detected; patients were diagnosed with hypopharyngeal carcinoma for the first time without complication of other malignant tumors; none preoperative radiotherapy, chemotherapy or targeted therapy was received. The collection process followed the Declaration of Helsinki and was approved by the Ethics Committee of the First Affiliated Hospital of Chongqing Medical University. Some specimens were immediately frozen in liquid nitrogen and stored at – 80 °C, others were immersed in formalin and then embedded in paraffin. The clinical characteristics of patients are shown in Table 1.

**Table 1:** The clinicopathological features of patients with hypopharyngeal carcinoma

Features	Variables	No. (%)
Age	< 60	39 (34.5)
	≥ 60	74 (65.5)
Smoking	Yes	103 (91.2)
	No	10 (8.8)
Gender	Male	108 (95.6)
	Female	5 (4.4)
Pathological stage	Early (T1–T2)	25 (22.1)
	Advanced (T3–T4)	88 (77.9)
Lymphatic metastasis	Presence	82 (72.6)
	Absence	31 (27.4)
Pathological differentiation	Low	37 (32.7)
	Moderate and high	76 (67.3)
Extracapsular spread	Yes	59 (52.2)
	No	54 (47.8)

### Transcriptome sequencing and qRT-PCR

Total RNA was extracted from the 10 cases of hypopharyngeal carcinoma primary tumor tissue (5 patients have lymphatic metastasis and 5 patients do not have lymphatic metastasis) using the RNA extraction kit (Takara, Dalian, China), and then sent to Jingzhou Gene Technology Limited Company (Shanghai, China) for illumina dual-terminal transcriptome sequencing. A threshold of 1.5 for the log2 fold change(FC) was set, and the edge software package was used to analyze the differentially expressed genes(|log2FC|> 1.5, *P* < 0.05). Then the volcano map and heat map of differential genes were plotted. Gene Ontology (GO) terms and Kyoto Encyclopedia of Genes and Genomes (KEGG) pathways were identified with a strict cutoff (false discovery rate (FDR) < 0.05). After the differential gene RAF1 was screened by transcriptome sequencing, the RNA was reverse transcribed into cDNA using PrimeScript RT reagent Kit (Takara, Dalian, China). Then, qRT-PCR was run by using SYBR primescript RT-PCR Kit (Takara, Dalian, China) and specific primers for GAPDH and RAF1. The primer sequences were as follows: GAPDH (sense): CAGCGACACCCACTCCTC; GAPDH (antisense): TGAGGTCCACCACCCTGT; RAF1 (sense): GGGAGCTTGGAAGACGATCAG; RAF1(antisense): ACACGGATAGTGTTGCTTGTC. After the reactions were complete, the CT values were determined by setting a fixed threshold.The relative amounts of RAF1 mRNA were normalized to GAPDH using a 2^−ΔΔCt^ calculation method.

### Protein isolation and WB

The total protein extraction kit (KeyGen BioTECH, Jiangsu, China) was used to isolate proteins from the above 10 patients. The protein lysates were separated by 10% sodium dodecyl sulphate—polyacrylamide gels (SDS-PAGE) (Beyotime, Shanghai, China), and then transferred onto polyvinylidene difluoride (PVDF) membranes (Beyotime, Shanghai, China). Primarry antibodies against RAF1, phospho-RAF1 (p-RAF1) and GAPDH were purchased from Abcam (ab-137435, ab-60985, ab-181602; Abcam, Cambridge, UK) and were diluted at 1:1000, 1:1000 and 1:3000. The secondary antibody goat anti-rabbit IgG (Beyotime, Shanghai, China) was diluted at 1:5000. The Efficient chemiluminescence (ECL) kit (Thermo, Shanghai, China) was utilized to detect horseradish peroxidase (HRP) and its products.

### Immunohistochemistry (IHC)

IHC was performed on 4 μm sections of paraffin embedded tissues previously prepared, including 113 cases of tumor tissues from hypopharyngeal carcinoma patients (82 patients have lymphatic metastasis and 31 patients do not have lymphatic metastasis). After antigen retrieval and peroxidase block, slides were incubated with primary antibody (ab-60985, Abcam, Cambridge, UK) which was diluted at 1:200. Then, slides were treated with boost IHC detection reagent (ZSGB-BIO, Guangzhou, China). Finally, slides were stained with diaminobenzidine (DAB) and counterstained with hematoxylin. The immunohistochemistry results were analyzed separately by two experienced pathologists and scored considering both the intensity of staining and the proportion of tumor cells with an unequivocal positive reaction. Slides which incubated with Phosphate buffer saline (PBS) instead of primary antibody were selected as negative controls. According to the staining results, the expression was scored as either positive expression (stained brown, tumor cells ≥ 50%) or negative expression (stained yellow, tumor cells < 50% of cells).

### Clinical prognostic value analysis

According to the IHC results, Kaplan–Meier and log-rank test and COX’s univariate and multivariate analysis were used to examine the association among the clinicopathological features, and to evaluate independent risk factors of prognosis in hypopharyngeal carcinoma.

### Cell culture

The FADU cell line (human pharyngeal squamous cell line) was purchased from Center for Molecular and Cellular Sciences, Chinese Academy of Sciences (Shanghai, China). The SCC15 cell line (human tongue squamous cell line) was donated by Department of Oral and Maxillofacial Surgery, The First Affiliated Hospital of Chongqing Medical University. Cells were grown in dulbecco’s modified eagle medium (DMEM) high glucose medium (Gibco, Carlsbad, CA, USA) supplemented with 10% fetal bovine serum (FBS) (Gibco, Carlsbad, CA, USA) in a humidified incubator at 37 °C with 5% CO2.

### Lentivirus transfection

RAF1 was knocked down and overexpressed by transfection of FADU and SCC15 cell lines with lentivirus short hairpin RNA (shRNA) and overexpression vector. Lentiviruses were purchased from GeneChem (Shanghai, China), and the lentiviral composition was Ubi-firefly_Luciferase-IRES-Puromycin. The transfected cells were divided into four groups including sh-NC (knockdown negative control), sh-RAF1 (knockdown), vector (overexpression negative control) and lv-RAF1-OE (overexpression). FADU and SCC15 cells were seeded in 6-well plates and transfected using lentiviruses on the following day when the cells were approximately 50–60% confluent. The multiplicity of infection (MOI) of the cells was 10. After 6 h transfection, the cell medium was cultured in DMEM supplemented with 10% FBS for 48 h. Then, the cells were cultured in medium containing 2 μg/mL puromycin to obtain stably infected cells. Next, the cells were harvested for qRT-PCR and WB (methods were the same as above) to detect the expression of RAF1. Primarry antibodies against lymphangiogenic cytokines VEGF-C (ab83905, Abcam, Cambridge, UK, diluted at 1:1000), LYVE-1 (ab219556, Abcam, Cambridge, UK, diluted at 1:1000) and PROX-1 (ab199359, Abcam, Cambridge, UK, diluted at 1:1000) were added to WB.

### EdU proliferation assay

For EdU assay, FADU and SCC15 cells were seeded in 6-well plates after transfection. When the confluency of cells reached 80%, EdU assay kit (RiBoBio, Guangzhou, China) was used to determine the proliferation rate of the cells. The manufacturer’s instruction was followed except that the nucleus staining dye was changed from Hoechst 33,342 to DAPI (Beyotime, Shanghai, China). After staining, the cells were captured by inverted fluorescence microscope.

### Flow cytometry

For cell apoptosis assay, FADU and SCC15 cells were cultureed in 6-well plates until the confluency of cells reached 80–90%. Then, cells were labeled in a binding buffer containing annexin V-FITC/PI. The samples were analyzed by flow cytometry (Biosciences, CA, USA). For cell cycle assay, cells were trypsinized and fixed with 70% ice ethanol overnight at − 4 °C. Next day, the cells were treated with RNase A and propidium iodide and the cell cycle was measured by flow cytometry.

### Transwell migration and invasion assay

Cell migration assays were performed using 24-well plates with an 8-μm pore membrane (Corning, CA, USA). FADU cells were suspended in FBS-free DMEM medium and then added to the upper chamber (5 × 10^4^ cells/well). Meanwhile, DMEM medium plus 15% FBS was added to the lower compartment. The plates were incubated for 24 h in the incubator. After incubation, cells that had migrated to the lower surface of the filter membrane were fixed with 4% paraformaldehyde for 30 min at room temperature. The membrane was washed with PBS and stained with 0.5% crystal violet in methanol for 15 min at room temperature. Cells remaining on the upper surface of the filter membrane were gently scraped off with a cotton swab. The lower surfaces were captured by inverted fluorescence microscope, and the cells were counted blindly (five fields per chamber). In order to further evaluate cell invasion, the assay was repeated in a Transwell assay with Matrigel (Biosciences, MA, USA).

### Wound healing assay

FADU cells were seeded in 6-well plates (5 × 10^5^ cells/well) and cultured with DMEM medium supplemented with 10% FBS until the confluency of cells reached nearly 100%. The pipette tip was used to make 3–5 parallel scratches on the bottom of the Petri dish. Then the cells were washed with PBS and cultured with FBS-free medium in the incubator. The scratches were captured by inverted fluorescence microscope at 0 h, 24 h and 48 h. Meanwhile, the closure rate of wound was calculated.

### Establishment of foot-pad xenograft model in nude mice

Five-week-old male BALB/c-Nude mice were purchased from the HFK Bioscience Limited Company (Beijing, China) and maintained under specific pathogen-free conditions at Animal Center of Chongqing Medical University. Stably infected cells (including FADU-sh-NC, FADU-sh-RAF1, FADU-vector and FADU-lv-OE) were subcutaneously injected into foot-pad (1 × 10^6^ cells in 0.2 ml PBS per mouse) and the mice were divided into four groups (10 mice in each group). After 28 days, D-luciferin (Beyotime, Shanghai, China) was diluted with PBS to 15 mg/ ml and then injected intraperitoneally at a dose of 10 uL/g. Fluorescence imaging of nude mice was then performed. Next day, mice were sacrificed after injection of pentobarbital sodium. Primary tumor of foot-pad and metastatic lymph node were collected, and the volumes and weights of the tumors were measured. The volume of tumors were calculated by the formula: Tumor volume = [length * (width)^2^] * π/6 [45]. A portion of the tissues was used for protein and total RNA extraction, and the remaining tissue was fixed in 4% paraformaldehyde. Then, the expression of RAF1 was detected by qRT-PCR, WB and IHC refer to the above methods. All experiments on mice were approved by the Ethics Committee of the First Affiliated Hospital of Chongqing Medical University and performed in accordance with the U.K. Animals (Scientific Procedures) Act and the guidelines of the National Institutes of Health.

### Proteomic sequencing

Proteins from foot-pad primary tumors of 8 nude mice (4 mice injected with FADU-sh-NC cells and 4 mice injected with FADU-sh-RAF1 cells) were extracted, and then sent to Jingzhou Gene Technology Limited Company (Shanghai, China) for TMT labeled quantitative proteomics. A threshold of 0.5 for the log2 fold change(FC) was set, and the edge software package was used to analyze the differentially expressed proteins(|log2FC|> 0.5, *P* < 0.05). Then the volcano map and heat map of differential proteins were plotted. GO terms and KEGG pathways were identified with a strict cutoff (false discovery rate (FDR) < 0.05). The expression level of RAF1-correlated proteins was analyzed by NetworkAnalyst.

### In vitro experiment of LAGE1

Lentivirus transfection was used to knock down LAGE1. Lentiviruses (including sh-LAGE1 and sh-NC) were purchased from GeneChem (Shanghai, China), and the lentiviral vector was Ubi-firefly_Luciferase-IRES-Puromycin. Puromycin was used to successfully obtain stably infected cells. RAF1 inhibitor Sorafenib (MCE, CA, USA) was diluted at 25 μmol/L with PBS and added to medium. Then, the cells were harvested for qRT-PCR and WB (methods were the same as above) to detect the expression of LAGE1. The primary antibody against LAGE1 was purchased from Abcam (ab177947, diluted at 1:1000). Next, EdU proliferation assay, Flow cytometry, Wound Healing assay and Transwell migration and invasion assay were used to verify the effect of LAGE1 expression changes on cell function.

### In vivo experiment of LAGE1

Five-week-old male BALB/c-Nude mice were purchased and maintained under specific pathogen-free conditions. Stably infected cells were subcutaneously injected into foot-pad and Sorafenib was injected intraperitoneally at a dilution of 30 mg/kg with PBS. The mice were divided into four groups: sh-NC, sh-NC + Sorafenib, sh-LAGE1 and sh-LAGE1 + Sorafenib (10 mice in each group). Fluorescence imaging of nude mice was then performed after 28 days. Next day, mice were sacrificed after injection of pentobarbital sodium. Primary tumor of foot-pad and metastatic lymph node were collected, and the volumes and weights of the tumors were measured. Then, qRT-PCR, WB and IHC were performed to detect the expression of LAGE1 (methods were the same as above).

### Statistical analysis

All of the images of the WB assay, IHC assay, EdU assay, Flow cytometry, Transwell assay and animal experiments were representative of at least three independent experiments or staining results. The qRT-PCR assay was performed in triplicate, and each individual experiment was repeated several times. SPSS 22.0 and GraphPad Prism 5.0 were employed for statistical analysis. The results are presented as the means ± standard deviation (SD). Observed differences were considered statistically significant at *P* < 0.05 by using Student’s *t*-test or Chi-square test.

## Results

### RAF1 is a differential gene for lymphatic metastasis of hypopharyngeal cancer, and the up-regulation of RAF1 expression is an independent risk factor for prognosis of hypopharyngeal cancer

**Fig. 1:**
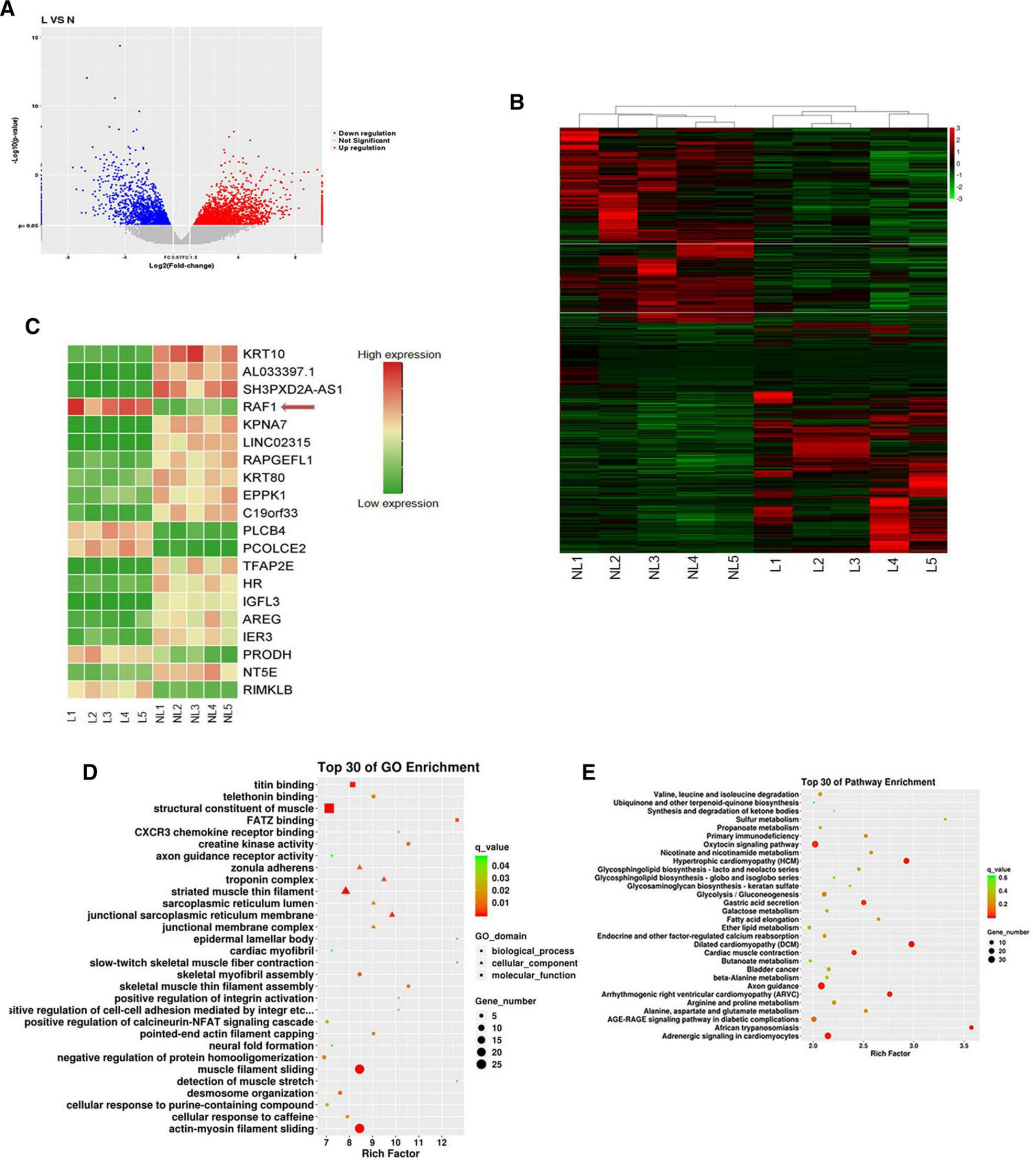
Transcriptome sequencing results and analysis. **A** Volcano map of differential genes, threshold of fold change (FC) was set as |log2FC|> 1.5, *P* < 0.05. **B** Heat map of differential genes. **C** Heat map of the top 20 differential genes, red arrow indicates that RAF1 is one of these genes. **D** GO terms analysis. **E** KEGG pathway enrichment analysis

**Fig. 2:**
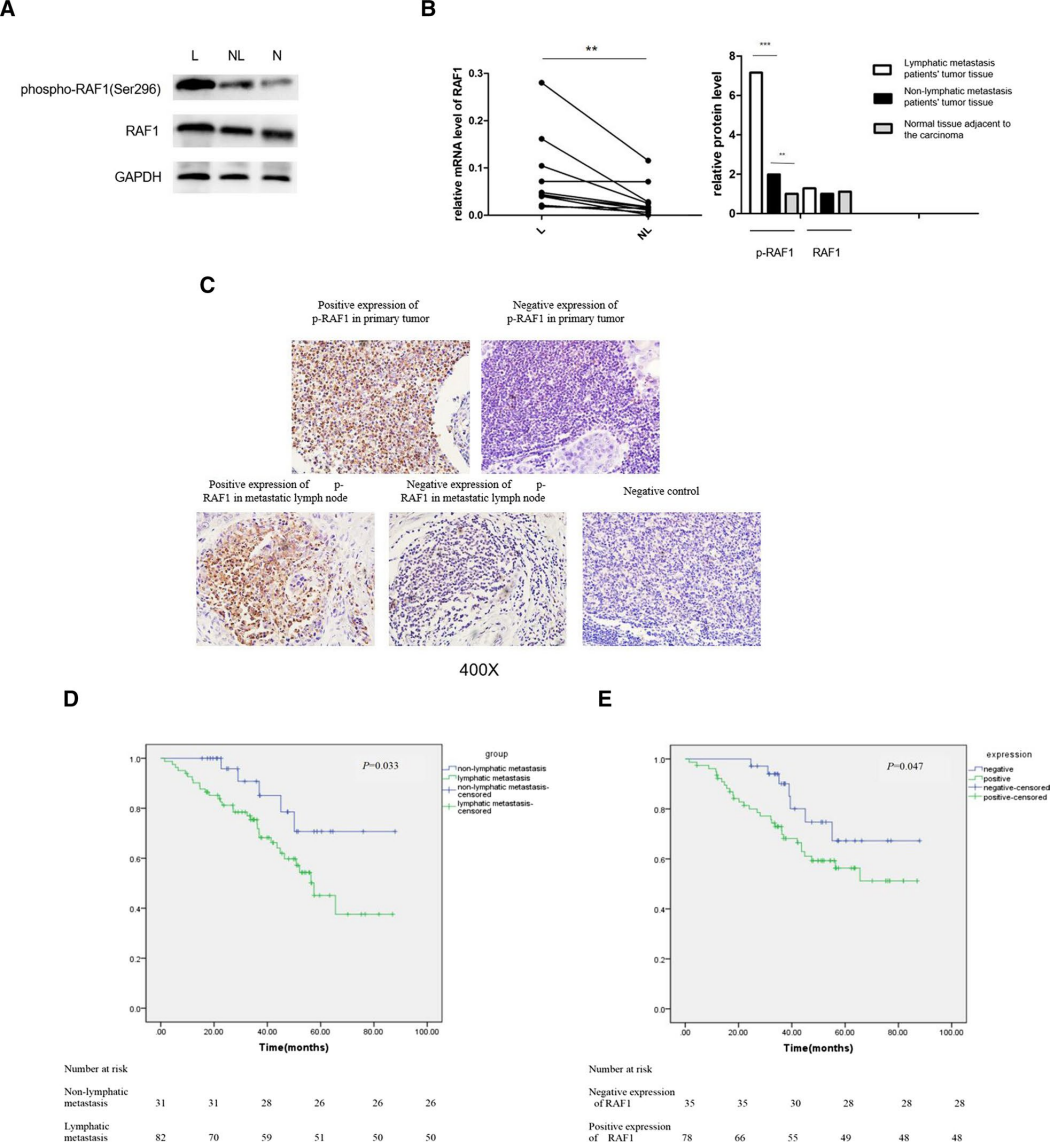
Expression and prognostic value analysis of RAF1 in clinical specimens. **A**–**B** qRT-PCR and WB assay of RAF1. **C** Immunohistochemical staining for RAF1. **D** Kaplan–meier survival curve of of risk factor for lymphatic metastasis. **E** Kaplan–meier survival curve of of risk factor for RAF1 expression. L, lymphatic metastasis patients' tumor tissue; NL, non-lymphatic metastasis patients’ tumor tissue; N, Normal tissue adjacent to the carcinoma. **P < 0.01, ***P < 0.001

Primary tumors from 5 patients with lymphatic metastasis and from another 5 patients without lymphatic metastasis were sent for transcriptome sequencing. A total of 2341 differential genes were screened based on the volcano map with a threshold of |log2FC|> 1.5, *P* < 0.05 (Fig. 1A). According to the heat map, we listed the top 20 genes with the most statistically significant differences in expression between the lymphatic and non-lymphatic groups, and RAF1 was one of them as indicated by the arrow (Fig. 1B and C). To further confirm the expression of RAF1 in clinical tumor tissue specimens, we assessed RAF1 mRNA and protein levels by qRT-PCR and WB assay. Results showed that in 10 pairs of hypopharyngeal carcinoma primary tumor tissues, the mRNA levels of RAF1 were significantly up-regulated in lymphatic metastasis group compared to non-lymphatic metastasis group (Fig. 2B). Meanwhile, the protein levels of phospho-RAF1 (p-RAF1) decreased sequentially in the lymphatic metastasis group, the non-lymphatic metastasis group and the normal tissue group (Fig. 2A and B). We increased the number of samples to 113 cases and confirmed the expression of RAF1 by IHC assay. It suggested that the positive expression rate of p-RAF1 was significantly higher in the group with lymphatic metastasis (66/82, 80.5%) than in the group without lymphatic metastasis (9/31, 29.0%) (Fig. 2C and Table 2). In addition, the expression of p-RAF1 was positive in most of the metastatic lymph nodes (71/95, 74.7%). Kaplan–Meier & log-rank test and COX’s analysis indicated that RAF1 positive expression and lymphatic metastasis are both independent prognostic risk factors for hypopharyngeal carcinoma (Fig. 2D, E and Table 3). These results suggest that RAF1 expression level may be directly related to lymphatic metastasis.

**Table 2:** The immunohistochemical expression of RAF1 in clinical specimens of patients

Tissue	Number of patients	Expression of p-RAF1		*P* Value*
		Positive	Negative	
Primary tumor in patients with lymphatic metastasis	82	66	16	*P* < 0.001
Primary tumor in patients without lymphatic metastasis	31	9	22	
Metastatic lymph node in patients	95	71	24	

^*^P values are from χ2 test or Fisher’s exact test and were statistically significant when < 0.05

**Table 3:** Univariate and multivariate Cox regression analyses of overall survival in hypopharyngeal carcinoma patients

Variable	Univariate analyses			Multivariate analyses		
	HR	95%CI	P*	HR	95%CI	P*
Age	1.738	0.791–3.576	0.341			
Smoking	1.797	0.994–5.260	1.109			
Gender	0.037	0.014–12.581	0.803			
Pathological stage	1.429	0.056–3.292	0.094			
Lymphatic metastasis	3.155	1.782–6.191	0.027	2.409	0.975–4.722	0.038
Pathological differentiation	1.126	0.603–2.797	0.062			
Expression of RAF1	1.894	1.069–4.467	0.033	1.153	0.776–3.291	0.048
Extracapsular spread	4.512	2.607–7.335	0.045	3.731	2.240–5.699	0.070

^*^P values are from Cox analyses were statistically significant when < 0.05

### Down-regulation of RAF1 inhibits the proliferation, migration and invasion of FADU and SCC15 cells, and promoted cell apoptosis

**Fig. 3:**
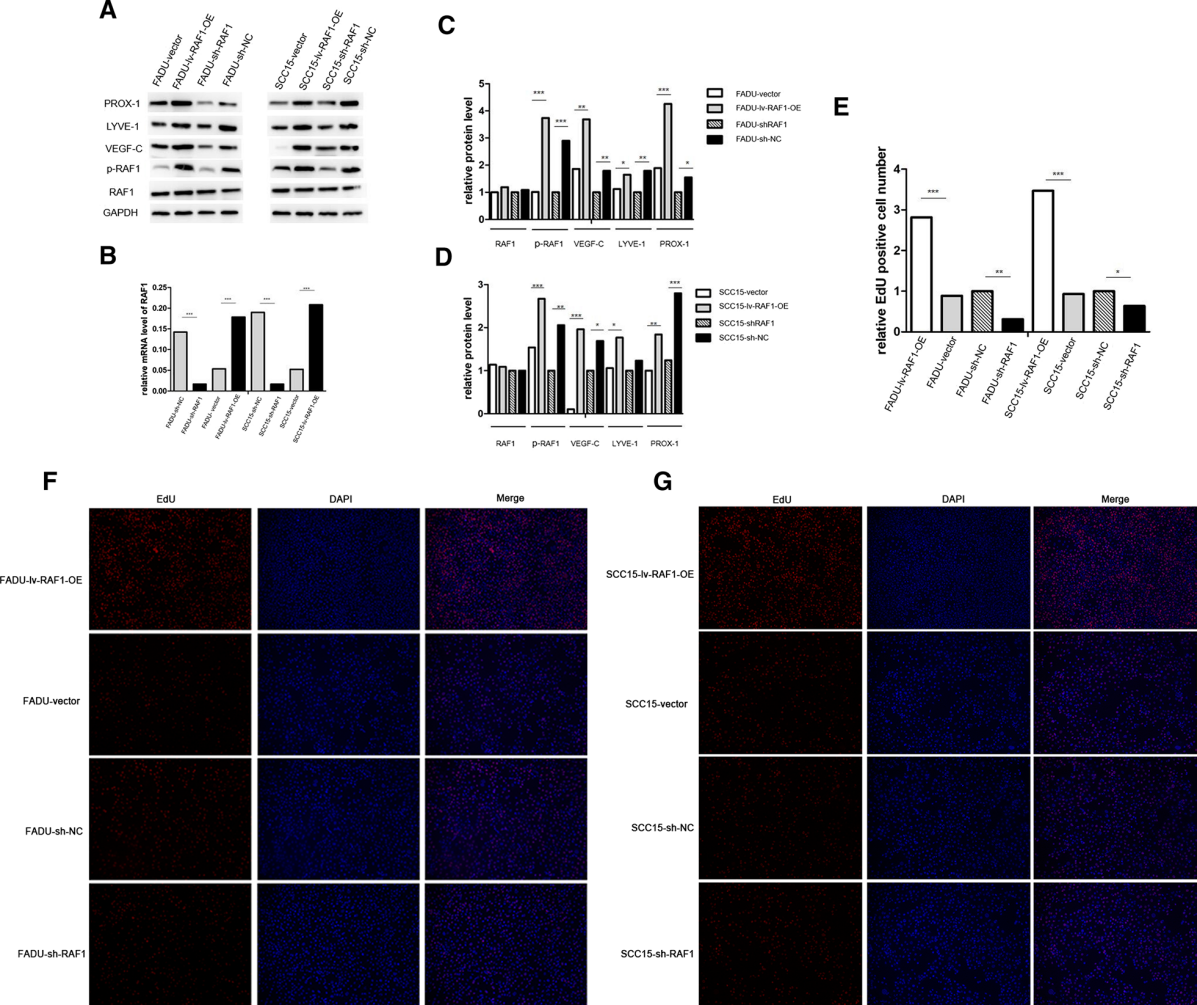
Expression of RAF1 and lymphangiogenic cytokines in cell lines and cell proliferation assay. **A**–**D** qRT-PCR and WB assay of RAF1 and lymphangiogenic cytokines. **F** EdU proliferation assay in FADU cell line. **G** EdU proliferation assay in SCC15 cell line. **E** Results analysis of EdU assay. Abbreviations: sh-RNA, short hairpin RNA; NC, negative control; lv, lentivirus; OE, overexpression. *P < 0.05, **P < 0.01, ***P < 0.001

**Fig. 4:**
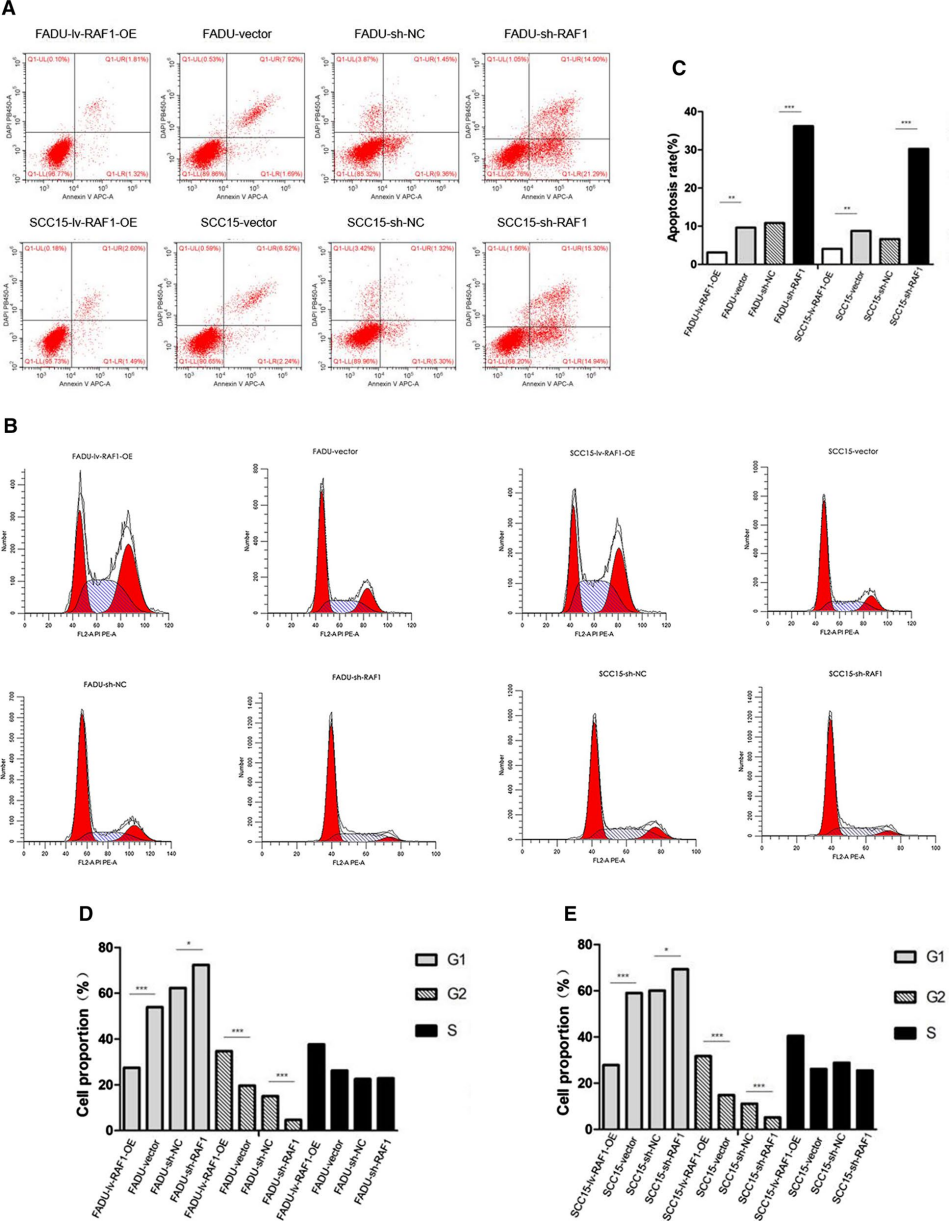
Flow cytometry assay. **A** Apoptosis assay in FADU and SCC15 cell lines. **B** Cell cycle assay in FADU and SCC15 cell lines. **C** Results analysis of Apoptosis assay; **D**, **E** Results analysis of Cell cycle assay. Abbreviations: sh-RNA, short hairpin RNA; NC, negative control; lv, lentivirus; OE, overexpression. *P < 0.05, **P < 0.01, ***P < 0.001

**Fig. 5:**
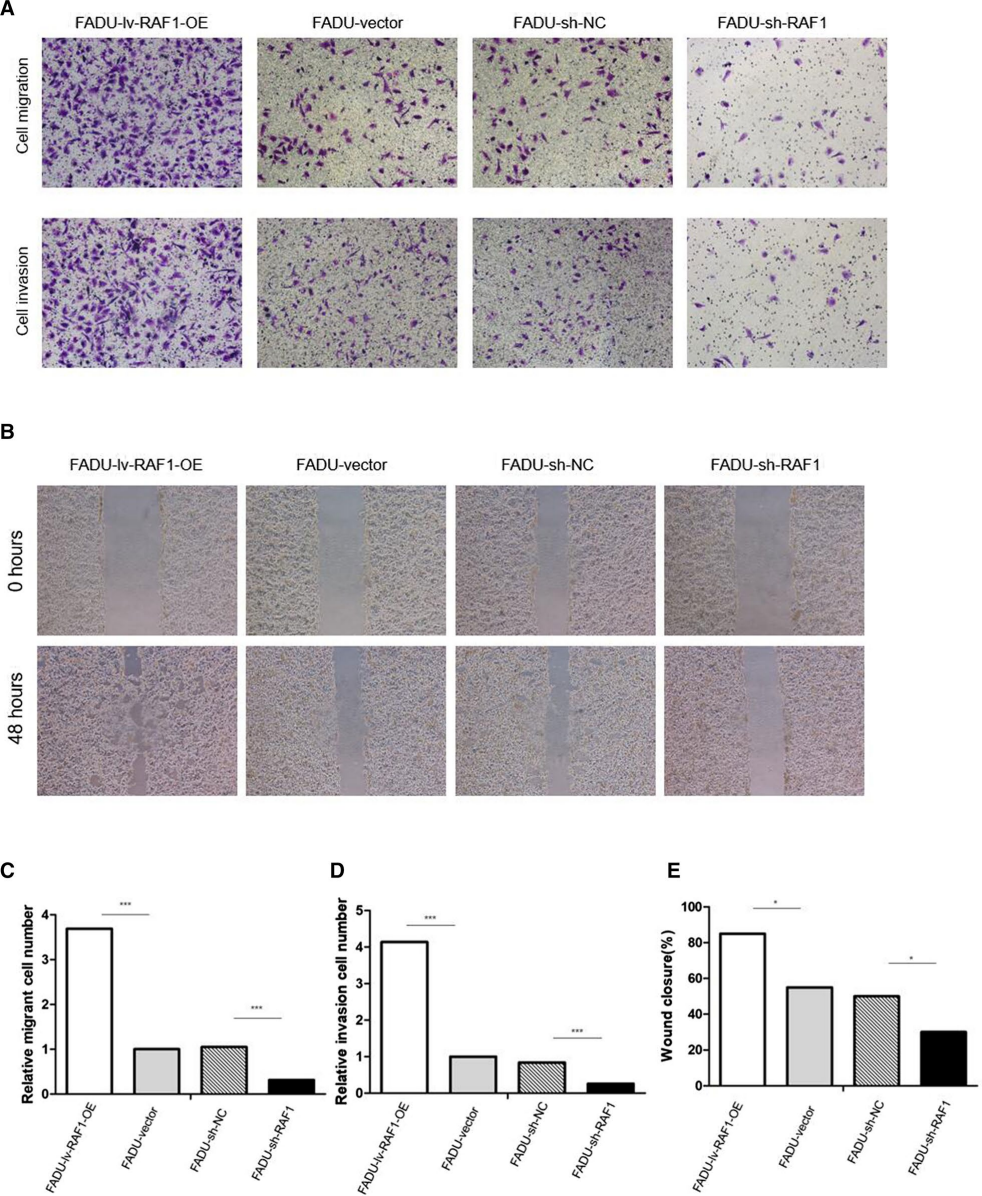
Transwell assay and Wound Healing assay. **A** Cell migration and invasion assay in FADU cell line. **B** Wound Healing assay in FADU cell line; **C**, **D** Results analysis of Transwell assay. **E** Results analysis of Wound Healing assay. Abbreviations: sh-RNA, short hairpin RNA; NC, negative control; lv, lentivirus; OE, overexpression. *P < 0.05, **P < 0.01, ***P < 0.001

RAF1 was knocked down and overexpressed by transfection of FADU and SCC15 cell lines with lentivirus shRNA and overexpression vector. According to qRT-PCR and WB assay (Fig. 3A–D), the mRNA and protein levels of RAF1/p-RAF1 were significantly decreased in knockdown group (sh-RAF1) and increased in overexpression group (lv-RAF1-OE) compared to their negative control groups (sh-NC and vector). In addition, the protein levels of lymphangiogenic cytokines (including VEGF-C, LYVE-1 and PROX-1) were consistent with the expression trend of p-RAF1 (Fig. 3A, C and D). It suggested that the expression of RAF1 might be related to lymphangiogenesis. The results of EdU assay indicated that down-regulation of RAF1 expression can decrease the proliferation ability of cells (Fig. 3E, F and G). Due to flow cytometry assay, the apoptosis rate was significantly reduced in RAF1 overexpression group and increased in knockdown group compared to the negative control groups (Fig. 4A and C). In RAF1 knockdown group, the proportion of G1 phase cells increased, while G2 phase cells decreased. Meanwhile, the overexpression group showed the opposite trend (Fig. 4B, D and E). Due to the weak invasion and migration ability of SCC15 cell line, the experimental results were not ideal, only FADU cell line was used in the Transwell assay and Wound Healing assay. The results showed that RAF1 knockdown significantly reduced the cell migration and invasion ability, while RAF1 overexpression greatly enhanced the cell migration and invasion ability (Fig. 5A–E). In conclusion, reduced expression of RAF1 can significantly inhibit the proliferation, migration and invasion of tumor cells, and promote the apoptosis of tumor cells.

### Up-regulation of RAF1 can promote tumor growth and lymphatic metastasis

**Fig. 6:**
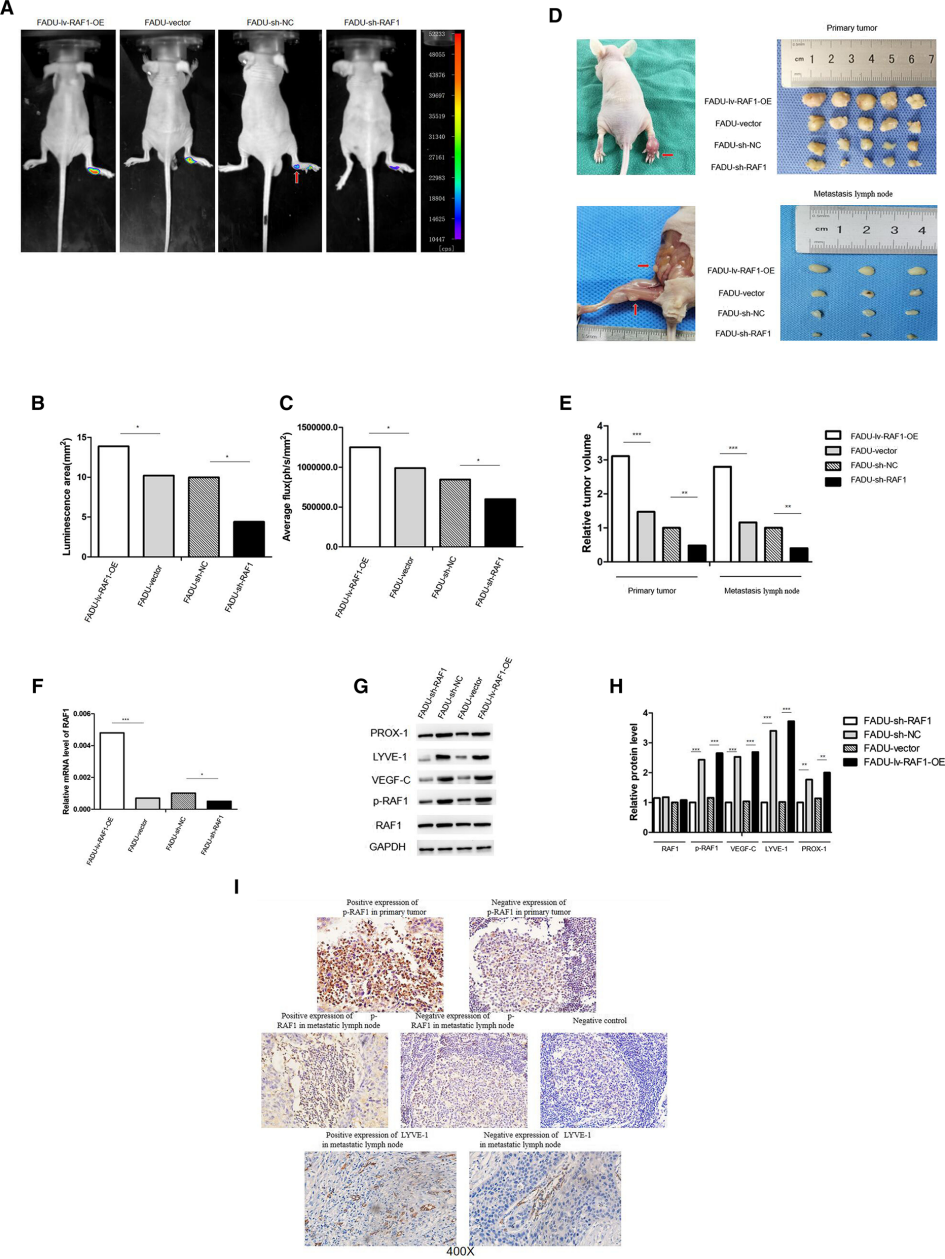
RAF1 regulates tumor growth and lymphatic metastasis in vivo. **A** Fluorescence imaging of nude mice, red arrow indicates suspected lymphatic metastasis. **B**, **C** Luminescence area and photon flux analysis. **D** Anatomy of foot-pad primary tumors and metastatic lymph nodes in nude mice, red arrow indicates metastatic lymph nodes; **E** Measurements of primary tumors and lymph nodes volume. **F**–**H** qRT-PCR and WB assay of RAF1 and lymphangiogenic cytokines. **I** Immunohistochemical staining for RAF1 and LYVE-1. Abbreviations: sh-RNA, short hairpin RNA; NC, negative control; lv, lentivirus; OE, overexpression. *P < 0.05, **P < 0.01, ***P < 0.001

We constructed a nude mouse xenograft model by injecting stably infected FADU cells into the foot-pad. Nude mice were divided into 4 groups according to the type of injected cells: FADU-sh-NC, FADU-sh-RAF1, FADU-vector and FADU-lv-RAF1-OE (10 mice in each group). Fluorescence imaging was performed after 28 days of feeding in specific pathogen-free conditions (Fig. 6A). Results analysis showed that luminescence area and average photon flux were reduced in sh-NC group and increased in lv-RAF1-OE group compared to their control group (Fig. 6B and C). After the mice were anesthetized and sacrificed, primary tumors of foot-pad and metastatic lymph nodes were harvested (Fig. 6D). Meanwhile, we measured the volumes and weights of the tumors (Fig. 6E) and counted the number of metastatic lymph nodes in each group (Table 4). The results indicated that in the sh-RAF1 group, the growth of primary tumor and lymph nodes was slower than that in the sh-NC group, and the incidence of lymphatic metastasis was lower. On the contrary, in the lv-RAF1-OE group, tumor growth was faster than in the control group, and the incidence of lymphatic metastasis was higher. RAF1 expression in primary tumors and lymph nodes was subsequently validated. According to qRT-PCR and WB assay (Fig. 6F, G and H), the mRNA and protein levels of RAF1/p-RAF1 were significantly decreased in sh-RAF1 group and increased in lv-RAF1-OE group compared to their negative control groups. The protein levels of lymphangiogenic cytokines (including VEGF-C, LYVE-1 and PROX-1) were consistent with the expression trend of p-RAF1. Furthermore, the result of IHC suggested that the positive expression rate of p-RAF1 was higher in lv-RAF1-OE group (80.0% in primary tumors, 71.4% in metastatic lymph nodes) than in vector group (50.0% in primary tumors, 25.0% in metastatic lymph nodes), and was lower in sh-RAF1 group (20.0% in primary tumors, 0% in metastatic lymph nodes) than in sh-NC group (60.0% in primary tumors, 40.0% in metastatic lymph nodes) (Fig. 6I and Table 4). In addition, the expression of lymphangiogenic cytokine LYVE-1 was verified in metastatic lymph nodes. Result showed that the positive rate decreased sequentially in lv-RAF1-OE group, vector group, sh-NC group and sh-RAF1 group (Fig. 6I and Table 5). It suggested that lymphangiogenesis might decrease with RAF1 down-regulation. In general, in vivo results indicated that increased RAF1 expression promoted both tumor growth and lymphatic metastasis.

**Table 4:** The immunohistochemical expression of RAF1 in specimens of nude mice

Tissue	Number of nude mice	Expression of p-RAF1	
		Positive	Negative
Primary tumor in lv-RAF1-OE group	10	8	2
Primary tumor in vector group	10	5	5
Primary tumor in sh-NC group	10	6	4
Primary tumor in sh-RAF1 group	10	2	8
Metastatic lymph node in lv-RAF1-OE group	7	5	2
Metastatic lymph node in vector group	4	1	3
Metastatic lymph node in sh-NC group	5	2	3
Metastatic lymph node in sh-RAF1 group	2	0	2

**Table 5:** The immunohistochemical expression of LYVE-1 in lymph nodes of nude mice

Tissue	Number of nude mice	Expression of LYVE-1	
		Positive	Negative
Metastatic lymph node in lv-RAF1-OE group	7	7	0
Metastatic lymph node in vector group	4	3	1
Metastatic lymph node in sh-NC group	5	3	2
Metastatic lymph node in sh-RAF1 group	2	1	1

### RAF1 promotes lymphatic metastasis by targeting LAGE1

**Fig. 7:**
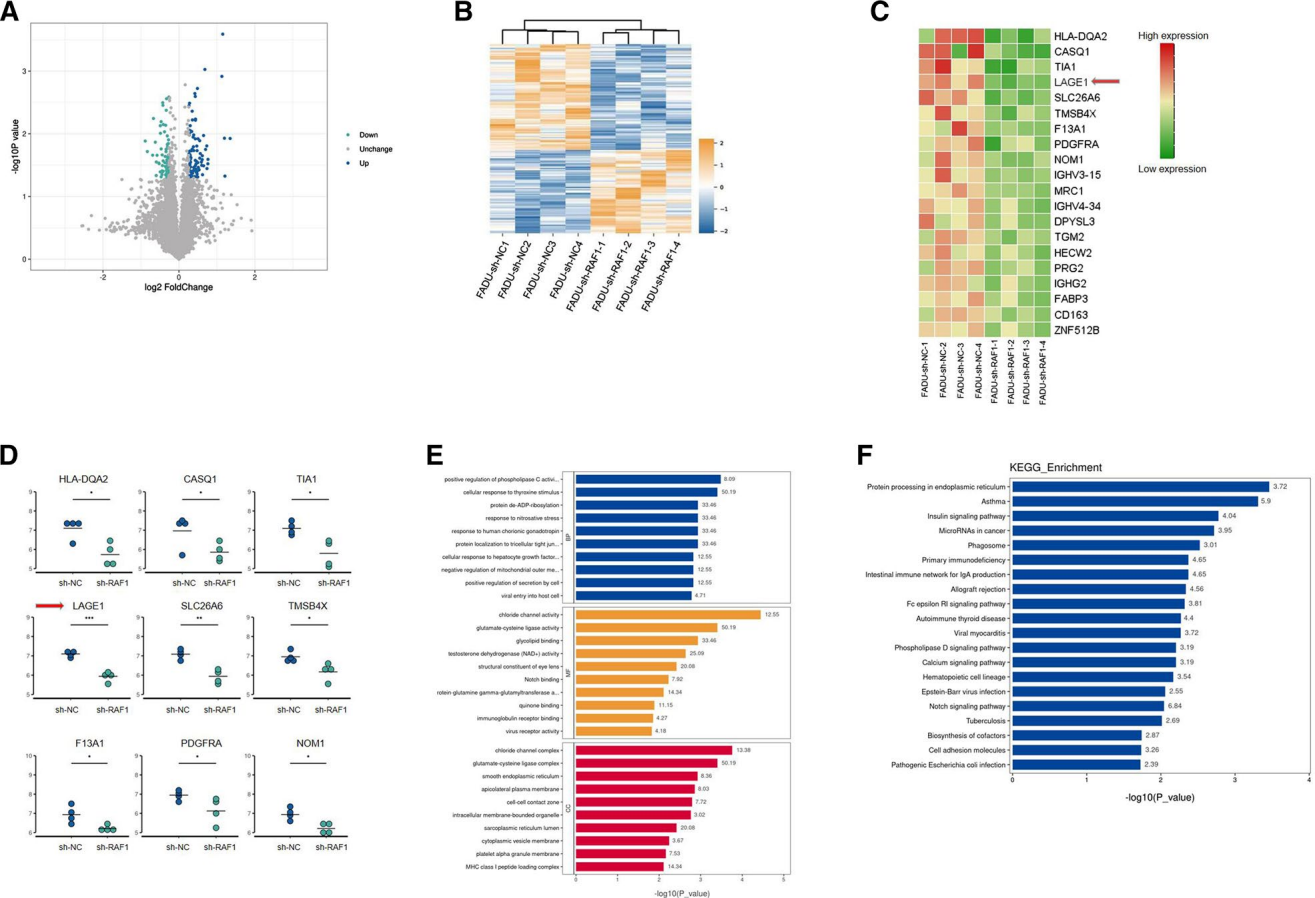
Proteomics sequencing results and analysis. **A** Volcano map of differential proteins, threshold of fold change (FC) was set as |log2FC|> 0.5, *P* < 0.05. **B** Heat map of differential proteins. **C** Heat map of the top 20 differential proteins, red arrow indicates that LAGE1 is one of these proteins. **D** The expression level of RAF1-correlated proteins was analyzed by NetworkAnalyst. Red arrow indicates that LAGE1 had the most significant expression difference between groups. **E** GO terms analysis; **F** KEGG pathway enrichment analysis. Abbreviations: sh-RNA, short hairpin RNA; NC, negative control. *P < 0.05, **P < 0.01, ***P < 0.001

**Fig. 8:**
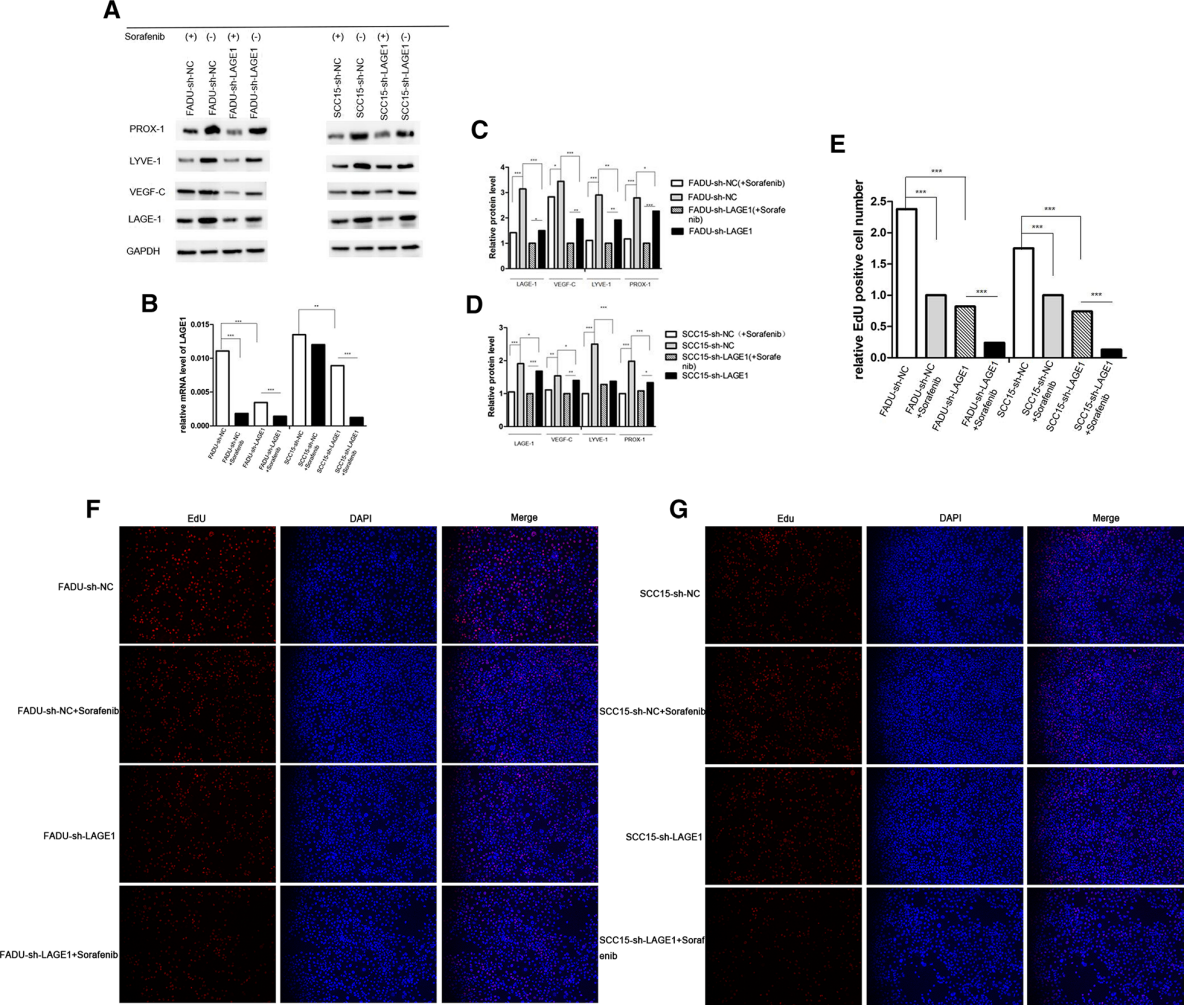
Expression of LAGE1 and lymphangiogenic cytokines in FADU cell line as well Edu proliferation assay. **A**–**D** qRT-PCR and WB assay of LAGE1 and lymphangiogenic cytokines. **F** EdU proliferation assay in FADU cell line. **G** EdU proliferation assay in SCC15 cell line. **E** Results analysis of EdU assay. Abbreviations: sh-RNA, short hairpin RNA; NC, negative control; (+), addition Sorafinib; (−) without Sorafinib. *P < 0.05, **P < 0.01, ***P < 0.00

**Fig. 9:**
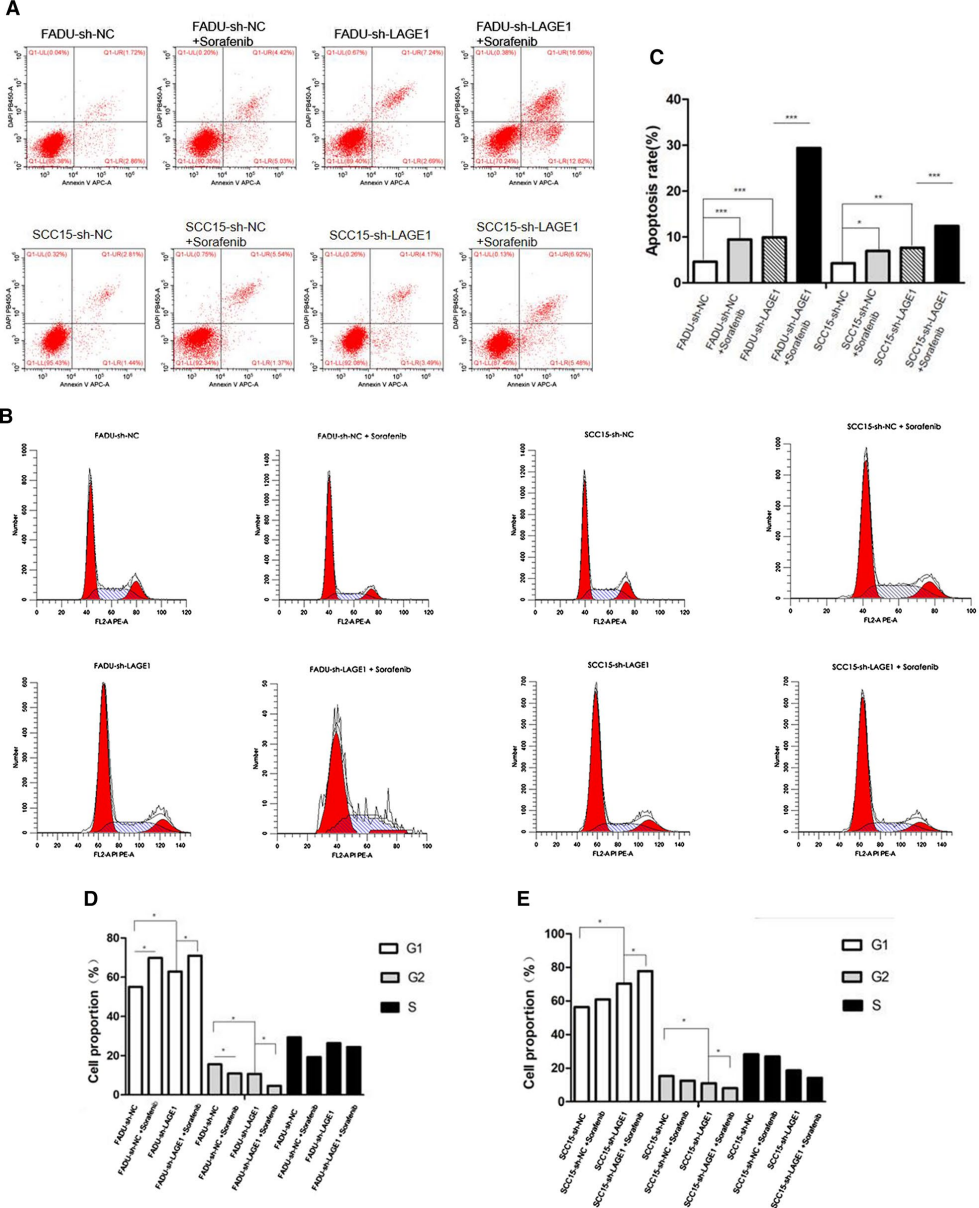
Flow cytometry assay. **A** Apoptosis assay in FADU and SCC15 cell lines. **B** Cell cycle assay in FADU and SCC15 cell lines. **C** Results analysis of Apoptosis assay. **D**, **E** Results analysis of Cell cycle assay. Abbreviations: sh-RNA, short hairpin RNA; NC, negative control. *P < 0.05, **P < 0.01, ***P < 0.001

**Fig. 10:**
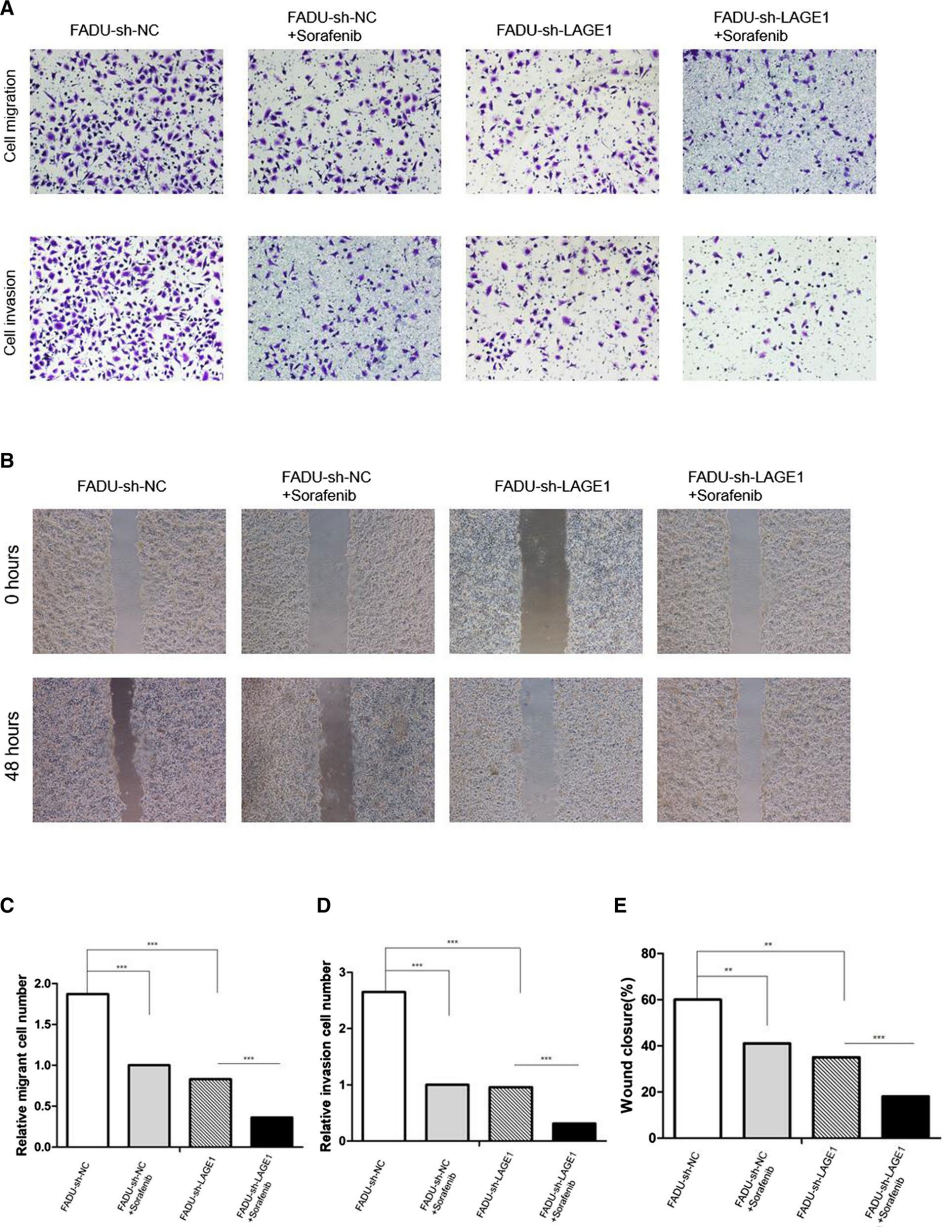
Transwell assay and Wound Healing assay. **A** Cell migration and invasion assay in FADU cell line. **B** Wound Healing assay in FADU cell line. **C**, **D** Results analysis of Transwell assay. **E** Results analysis of Wound Healing assay. Abbreviations: sh-RNA, short hairpin RNA; NC, negative control. *P < 0.05, **P < 0.01, ***P < 0.001

**Fig. 11:**
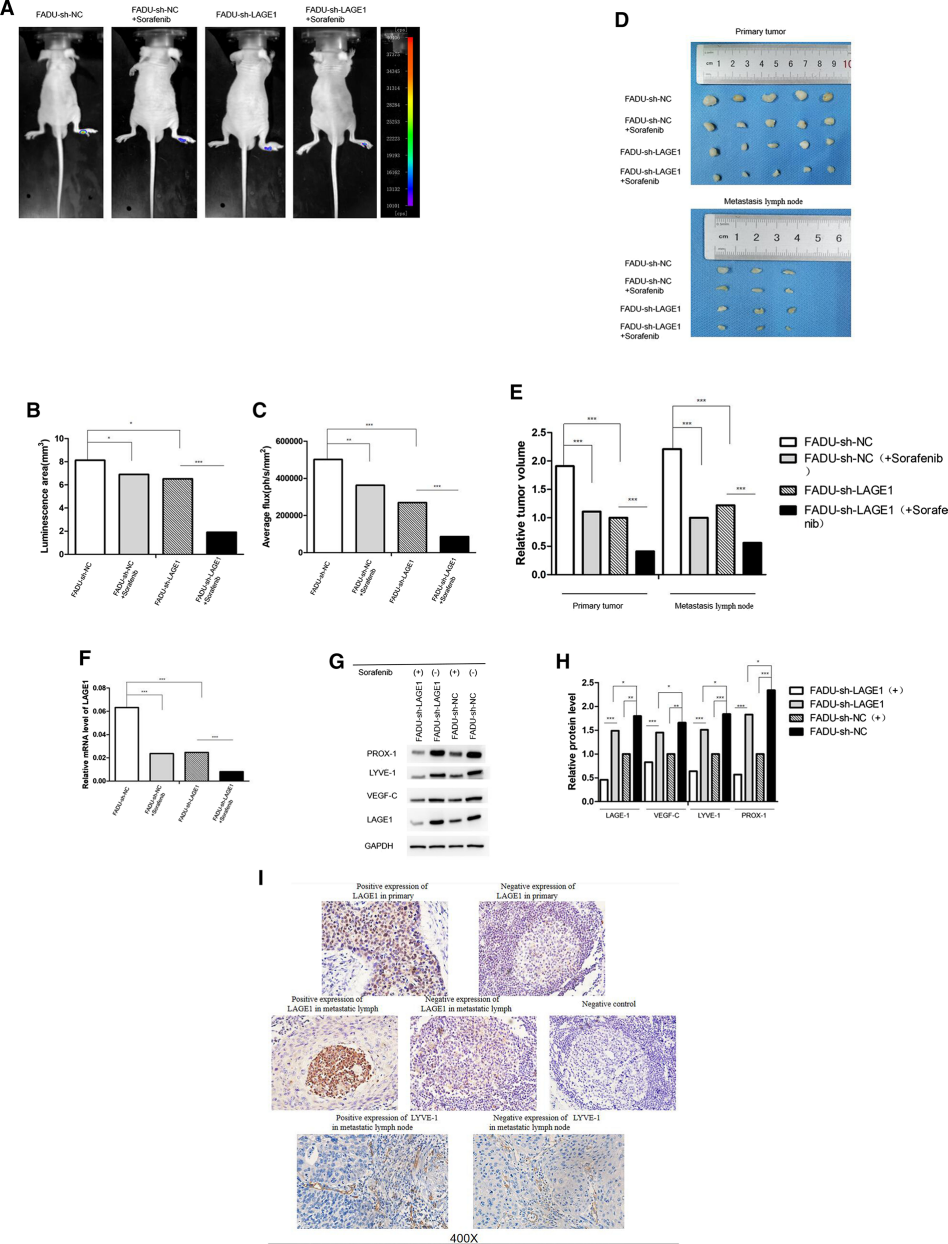
LAGE1 and Sorafenib regulate tumor growth and lymphatic metastasis in vivo. **A** Fluorescence imaging of nude mice. **B**, **C** Luminescence area and photon flux analysis. **D** Anatomy of foot-pad primary tumors and metastatic lymph nodes in nude mice. **E** Measurements of primary tumors and lymph nodes volume. **F**–**H** qRT-PCR and WB assay of LAGE1 and lymphangiogenic cytokines. **I** Immunohistochemical staining for LAGE1 and LYVE-1. Abbreviations: sh-RNA, short hairpin RNA; NC, negative control; (+), addition Sorafinib; (−) without Sorafinib. *P < 0.05, **P < 0.01, ***P < 0.001

Primary tumors from 4 mice in sh-NC group and from another 4 mice in sh-RAF1 group were sent for proteomics sequencing. A total of 159 differential proteins were screened based on the volcano map with a threshold of |log2FC|> 0.5, *P* < 0.05 (Fig. 7A). According to the heat map, we listed the top 20 proteins with the most statistically significant differences in expression between sh-NC group and sh-RAF1 group groups, and RAF1 was one of them as indicated by the arrow (Fig. 7B and C). The expression level of RAF1-correlated proteins was analyzed by NetworkAnalyst. Results showed that LAGE1 had the most significant expression difference between groups (Fig. 7D). Then LAGE1 was knocked down by transfection of FADU and SCC15 cell lines with lentivirus shRNA. Meanwhile, RAF1 inhibitor Sorafenib was added to divide the transfected cells into 4 groups: sh-NC, sh-LAGE1, sh-NC + Sorafenib and sh-LAGE1 + Sorafenib. The results of qRT-PCR and WB showed that the mRNA and protein levels were significantly decreased after LAGE1 knockdown, while the mRNA and protein levels were further decreased after Sorafenib addition (Fig. 8A–D). The results of EdU assay suggested that down-regulation of LAGE1 could significantly inhibit cell proliferation, and the addition of sorafenib could aggravate this trend (Fig. 8E, F and G). According to Flow cytometry, we found that decreased LAGE1 led to increased apoptosis, while the proportion of G1 phase cells increased and that of G2 phase cells decreased. Sorafenib made the difference in apoptosis and cell cycle even more significant (Fig. 9A–E). In Transwell assay and Wound Healing assay, down-regulation of LAGE1 significantly inhibited cell migration and invasion, while Sorafenib further inhibited these functions (Fig. 10A–E). Above in vitro experiments proved that LAGE1 down-regulation could inhibit the proliferation, migration and invasion of tumor cells and promote apoptosis, while RAF1 inhibitor Sorafenib could further enhance this trend. It suggested that the effects of LAGE1 on cell function might be regulated by RAF1. In vivo experiment, we injected stably infected FADU cells into foot-pad subcutaneously and injected sorafenib intraperitoneally to divided mice into same groups as in vitro experiment. Fluorescence imaging results showed that luminescence area and average photon flux were reduced when LAGE1 was down-regulated, and fluorescence indexes decreased more obviously after the addition of Sorafenib (Fig. 11A–C). Measurements of primary tumors and lymph nodes harvested from the mice suggested that decreased LAGE1 inhibited tumor growth, and sorafenib helped to aggravate this trend (Fig. 11D and E). According to qRT-PCR and WB assay, mRNA and protein levels were significantly decreased after LAGE1 knockdown, while the mRNA and protein levels were further decreased after Sorafenib addition. Meanwhile, lymphangiogenic cytokines (including VEGF-C, LYVE-1 and PROX-1) also showed the same protein expression trend (Fig.11F–H)). Moreover, the result of IHC indicated that the positive expression rate of LAGE1 was higher in sh-NC group than in sh-LAGE1 group. After sorafenib injection, the positive rate was further reduced (Fig. 11I and Table 6). In addition, the expression of LYVE-1 was verified in lymph nodes. Result showed that the positive rate decreased sequentially in sh-NC group, sh-NC + Sorafenib group, sh-LAGE1 group and sh-LAGE1 + Sorafenib group (Fig. 11I and Table 7). In conclusion, down-regulation of LAGE1 could also inhibit tumor growth and lymphatic metastasis, and this inhibition was more significant under RAF1 regulation. It suggested that RAF1 could modulate lymphatic metastasis by targeting LAGE1.

**Table 6:** The immunohistochemical expression of LAGE1 in specimens of nude mice

Tissue	Number of nude mice	Expression of LAGE1	
		Positive	Negative
Primary tumor in sh-NC group	10	7	3
Primary tumor in sh-NC + Sorafenib group	10	4	6
Primary tumor in sh-LAGE1 group	10	4	6
Primary tumor in sh-LAGE1 + Sorafenib group	10	2	8
Metastatic lymph node in sh-NC group	5	3	2
Metastatic lymph node in sh-NC + Sorafenib group	4	1	3
Metastatic lymph node in sh-LAGE1 group	3	1	2
Metastatic lymph node in sh-LAGE1 + Sorafenib group	3	0	3

**Table 7:** The immunohistochemical expression of LYVE-1 in lymph nodes of nude mice

Tissue	Number of nude mice	Expression of LYVE-1	
		Positive	Negative
Metastatic lymph node in sh-NC group	5	4	1
Metastatic lymph node in sh-NC + Sorafenib group	4	2	2
Metastatic lymph node in sh-LAGE1 group	3	1	2
Metastatic lymph node in sh-LAGE1 + Sorafenib group	3	0	3

## Discussion

The American Cancer Society estimated that HNSCC as the sixth most common malignancy worldwide, accounting for 5.3–7.1% of all systemic malignancies [46]. Hypopharyngeal carcinoma is one of the most malignant head and neck squamous cell carcinoma, accounting for about 0.8–1.5% of head and neck tumors. Due to the concealed anatomical structure of the hypopharynx, most patients are diagnosed in the advance clinical stage, and often accompanied by lymphatic metastasis. At present, hypopharyngeal carcinoma adopts the comprehensive treatment mode of surgery and adjuvant chemoradiotherapy, but the 5-year overall survival rate is still less than 50% [47, 48]. Lymphatic metastasis is an independent risk factor for prognosis of hypopharyngeal carcinoma and is main cause of treatment failure [49]. Lymphatic drainage is abundant in the hypopharyngeal region, and extensive anastomoses exist between parallel lymphatic systems. Abundant lymphatic network provides a certain anatomical basis for lymphatic metastasis of hypopharyngeal carcinoma, which makes hypopharyngeal carcinoma prone to lymphatic metastasis in early stage. It has been reported that the cervical lymph node metastasis rate of patients with hypopharyngeal carcinoma is 60–80%, while the occult lymph node metastasis rate of patients with cN0 stage is as high as 30% [50]. Lymphatic metastasis is the most common mode of tumor metastasis, which is closely related to tumor cells proliferation, migration, invasion, lymphangiogenic cytokines release, and lymphangiogenesis [18–20]. The cytokines VEGF-C, LYVE-1 and PROX-1 have been reported as lymphatic endothelial markers, which can directly lead to lymphangiogenesis [51–53]. Therefore, this study aims to provide new guidance for targeted therapy of tumors from the perspective of blocking signal pathways and antagonizing relevant effecting molecules by verifying the regulatory factors and mechanism of lymphatic metastasis in hypopharyngeal carcinoma.

Multi-omics sequencing is an accurate and efficient detection technique used to screen for differential genes and disease-related biomarkers [54]. This provides an important idea for us to search for the regulatory factors of lymphatic metastasis of hypopharyngeal carcinoma. Through transcriptomic sequencing and bioinformatics analysis of clinical specimens, as well as QRT-PCR and WB assay, RAF1 was proved to be a significantly differential gene between patients with and without lymphatic metastasis of hypopharyngeal carcinoma. RAF1 belongs to the RAF protein kinase family which contains A-RAF, B-RAF and RAF1 (C-RAF), and plays a signal conduction function in MAPK signaling pathway [55]. It has been reported in a variety of malignancies that RAF1 transmits extracellular signals into the nucleus through cell membrane receptors, thereby mediating the expression of intracellular specific proteins and participating in the regulation of cell proliferation, differentiation, apoptosis and autophagy [35, 56, 57]. In this study, lentivirus knockdown and overexpression of RAF1 were transfected into FADU and SCC15 cell lines, and it was proved that the downregulation of RAF1 could inhibit the proliferation, migration, invasion and promote apoptosis of tumor cells. In vivo experiments further demonstrated that down-regulation of RAF1 inhibited tumor growth and lymphatic metastasis. Therefore, it is of great significance to explore the intracellular proteins interacting with RAF1 to explain the mechanism of RAF1 regulating lymphatic metastasis.

To identify potential targets of RAF1, we sent mice foot-pad specimens for proteomic sequencing. Among the RAF1-correlated proteins analyzed by bioinformatics software, LAGE1 showed the most significant expression difference. The cancer–testis antigens contains 44 proteins (including LAGE1, MAGEC2, NY-ESO-1), the expression of which is characteristically restricted to cancer and the human germ line [58, 59]. Based on its immunogenicity and restricted tissue expression, LAGE1 seems an ideal target for active immunotherapies [60]. However, there are few studies on LAGE1 and tumor therapy in recent years, and the relationship between LAGE1 and lymphatic metastasis is rarely reported. In this study, in vivo and in vitro experiments proved that knockdown LAGE1 can also inhibit tumor development and lymphatic metastasis, which is consistent with the inhibitory effect of knockdown RAF1 as mentioned above. In addition, the RAF1 inhibitor Sorafenib could further down-regulate the expression of LAGE1, suggesting that LAGE1 may be simultaneously regulated by RAF1.

**Fig. 12:**
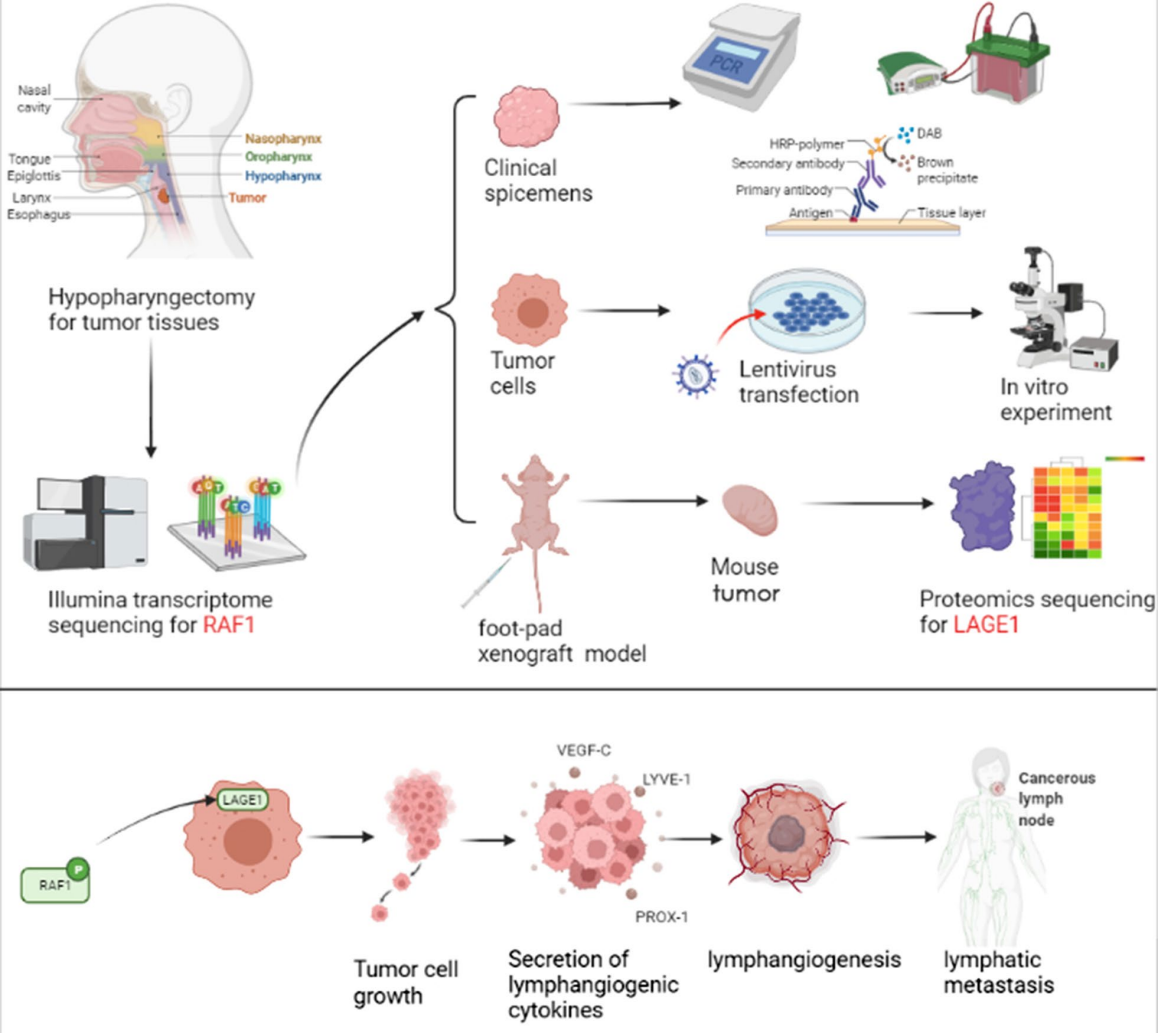
The experimental flow chart and hypothetical mechanism of the research

In this study, we demonstrated the hypothesis that down-regulation of extracellular protein kinase RAF1 leads to a decrease in intracellular LAGE1, which reduces the expression of lymphangiogenic cytokines (including VEGF-C, LYVE-1, and PROX-1), and ultimately inhibits lymphangiogenesis and lymphatic metastasis (Fig. 12). It suggests that RAF1 can promote lymphatic metastasis of hypopharyngeal carcinoma by regulating LAGE1, and provides a basis for the exploring of novel therapeutic target, and ultimately provides new guidance for the establishment of intelligent diagnosis and precise treatment of hypopharyngeal carcinoma. In fact, due to the limitation of time and conditions, the number of samples submitted for transcriptome and proteomics examination was not sufficient, so the sequencing results were inevitably subject to deviations due to individual differences. In addition, the sequencing results were not fully utilized, and there were many differential genes and proteins that we did not have time to verify. In further experiments, we plan to supplement metabolomic sequencing and single cell transcriptomic sequencing and expand the number of samples.

## Conclusions

In summary, this study verified for the first time that RAF1 can promote lymphatic metastasis of hypopharyngeal carcinoma by regulating LAGE1. We expect that the results of this study can provide new guidance for targeted therapy of tumors from the perspective of blocking signal pathways and antagonizing relevant effecting molecules.

## Data Availability

All data generated or analysed during this study are included in this published article.

## Abbreviations

HNSCC: Head and neck squamous cell carcinoma
LM: Lymphatic metastasis
RAF1: Ras-associated factor -1
LAGE1: L Antigen Family Member 1
qRT-PCR: Quantitative reverse transcription-polymerase chain reaction
WB: Western blotting
IHC: Immunohistochemical
FBS: Fetal bovine serum
sh-RNA: Short hairpin RNA
NC: Negative control
lv: Lentivirus
OE: Overexpression
